# Radiological concern for liposarcoma unmasked as extra-adrenal myelolipoma: A case report

**DOI:** 10.3892/mi.2026.318

**Published:** 2026-04-24

**Authors:** Shin Zaw, Lucas Douridas, Rodrigo Murillo Alvarez, Vania Zayat

**Affiliations:** 1 University of Central Florida/HCA Healthcare GME, Orlando, FL 34741, USA; 2 Department of Medicine, University of Central Florida College of Medicine, Orlando, FL 32827, USA; 3 Department of Pathology, Orlando Veterans Affairs Medical Center, Orlando, FL 32827, USA

**Keywords:** liposarcoma, myelolipoma, retroperitoneal mass, Cushing's disease, endocrine

## Abstract

Extra-adrenal myelolipomas (EAMLs) are rare, benign tumors composed of mature adipose tissue and trilineage hematopoiesis, most commonly arising in the retroperitoneum. When large or radiographically atypical, they can mimic malignant entities, such as well-differentiated liposarcomas, often necessitating tissue diagnosis. The present case report describes the case of a 79-year-old male patient with a history of pituitary adenoma treated with resection and radiation, resulting in secondary adrenal insufficiency and central hypothyroidism, who presented with progressive weakness, recurrent falls, and an unintentional weight loss of 18-20 lb. The laboratory evaluation did not reveal any notable findings, apart from known endocrine abnormalities. Non-contrast computed tomography (CT) revealed a heterogeneous, fat-containing left retroperitoneal mass, measuring 6.3x7.0x8.8 cm, with apparent ureteral encasement, concerning for liposarcoma. Surgical intervention was deferred due to comorbidities. A CT-guided biopsy demonstrated mature adipose tissue with trilineage hematopoietic elements, positive for CD45, MPO, CD71 and CD61, with flow cytometry excluding a clonal process, confirming EAML. EAMLs are exceedingly rare, with <100 cases reported, and definitive diagnosis relies on histopathology. The case described herein is notable for its association with remote Cushing's disease, supporting a potential link between chronic endocrine stimulation and EAML pathogenesis. EAML should be considered in the differential diagnosis of fat-containing retroperitoneal masses with atypical imaging features.

## Introduction

Myelolipomas are rare, benign tumors composed of mature fat and hematopoietic tissue, most commonly found in the adrenal glands, where they account for ~6-16% of all adrenal incidentalomas and represent the second most common adrenal lesion following adrenal adenomas (1). Their increased detection in recent decades is largely attributed to the widespread use of cross-sectional imaging for unrelated indications (2).

Extra-adrenal myelolipomas (EAMLs) are much less common and are typically discovered incidentally (3). They most commonly arise in the retroperitoneum, particularly the presacral region, although other documented sites include the mediastinum, liver, spleen, lungs, kidneys and paravertebral regions (3). When large or located in uncommon sites such as the retroperitoneum, they can mimic malignant tumors, such as well-differentiated liposarcoma on imaging, posing a significant diagnostic challenge (4).

The pathogenesis of myelolipomas is not yet fully understood. The proposed mechanisms include metaplastic transformation of adrenal cortical cells, mesenchymal stem cell differentiation and chronic stimulation by adrenocorticotropic hormone (ACTH), as suggested by their association with conditions, such as Cushing's disease and congenital adrenal hyperplasia (1,5). Although these lesions are benign, the presence of trilineage hematopoiesis can raise concern for underlying hematologic disease, prompting further evaluation (3).

The present case report describes the case of a male patient with a large retroperitoneal EAML initially suspected to be liposarcoma, ultimately diagnosed by core needle biopsy, with flow cytometry used to rule out a clonal process. The present case report highlights the diagnostic challenge posed by EAML, its potential association with prior endocrine disorders, and the importance of considering it in the differential for fat-containing retroperitoneal masses.

## Case report

A 79-year-old male patient with a complex medical history, including Cushing's disease due to pituitary adenoma, status post-transsphenoidal resection and radiation in 1999, with resulting secondary adrenal insufficiency and secondary hypothyroidism, diabetes, prostate cancer treated with radiation therapy and pellet implantation in 2001, with no known recurrence, presented to the Orlando Veterans Affairs Emergency Department (Orlando, FL, USA) in November, 2024 with progressive weakness, recurrent falls, and an unintentional weight loss of 18-20 lb over a period of several months. He denied having any gastrointestinal symptoms, such as dysphagia, nausea, or hematochezia, but reported having a decreased appetite and intermittent constipation.

**Figure 1:**
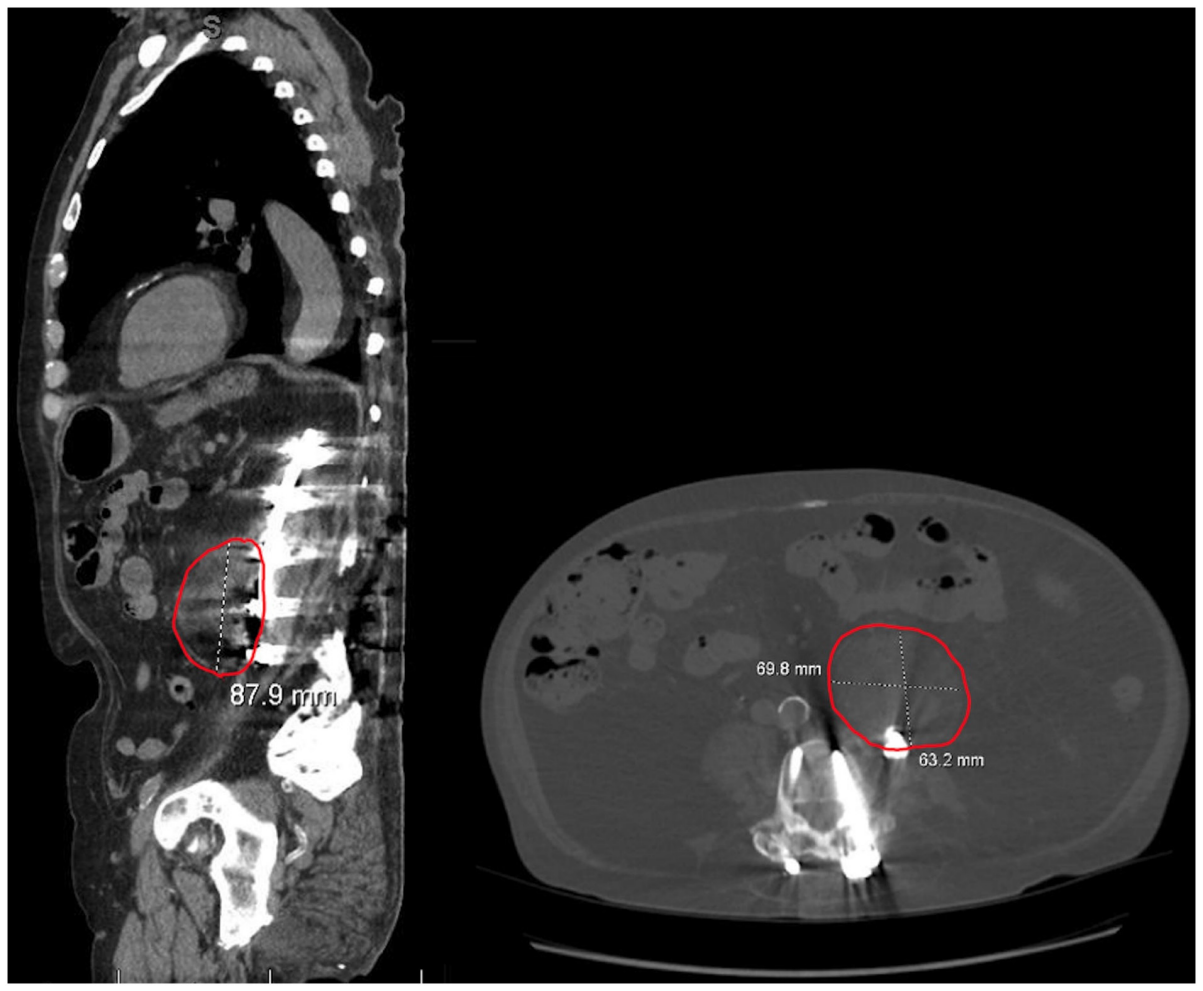
Sagittal (left panel) non-contrast computed tomography scan illustrating an 8.8-cm heterogeneous, fat-containing retroperitoneal mass. Axial (right panel) non-contrast computed tomography scan demonstrating the same lesion measuring ~6.9x6.3 cm. The size and appearance of the mass raised suspicion for liposarcoma prior to the biopsy confirmation of extra-adrenal myelolipoma.

Upon admission, the patient was normotensive and his complete blood count revealed a white blood cell count of 5.1x10^9^/l, hemoglobin of 14.1 g/dl, hematocrit of 43.4%, mean corpuscular volume of 94.8 fL and platelets of 208x10^9^/l. Hemoglobin A1c was 5.9%. Endocrine laboratory tests revealed a free T4 level of 1.6, while the morning cortisol level was 20.6 mcg/dl and the ACTH level was 11 pg/ml, which was consistent with his established profile on replacement therapy. Endocrinology was consulted. Follicle-stimulating hormone and luteinizing hormone levels had been within normal limits on outpatient laboratory tests at 1 month prior to admission. The analyses of vitamin B12 and folate levels, and the electrocardiogram (EKG) results did not reveal any notable findings. Brain magnetic resonance imaging (MRI) demonstrated a stable pituitary gland with no evidence of a recurrent tumor. Given his concerning constitutional symptoms, a malignancy workup was initiated. A non-contrast computed tomography (CT) scan of the abdomen and pelvis revealed a heterogeneous fat-containing left retroperitoneal mass measuring 6.3x7.0x8.8 cm (Fig. 1) with concerning features for liposarcoma, including the apparent encasement of the left ureter. Additional incidental findings included cystic pancreatic lesions and a right renal lesion, both recommended for further evaluation.

**Figure 2:**
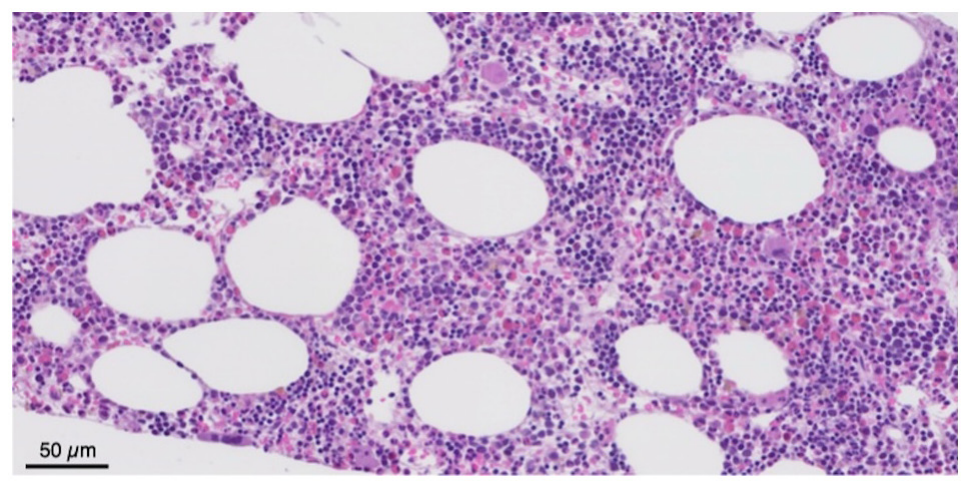
Hematoxylin and eosin staining at x200 magnification illustrating a well-circumscribed retroperitoneal mass composed of mature adipose tissue and trilineage hematopoietic elements, including erythroid, myeloid and megakaryocytic precursors, along with a small lymphoid aggregate.

**Figure 3:**
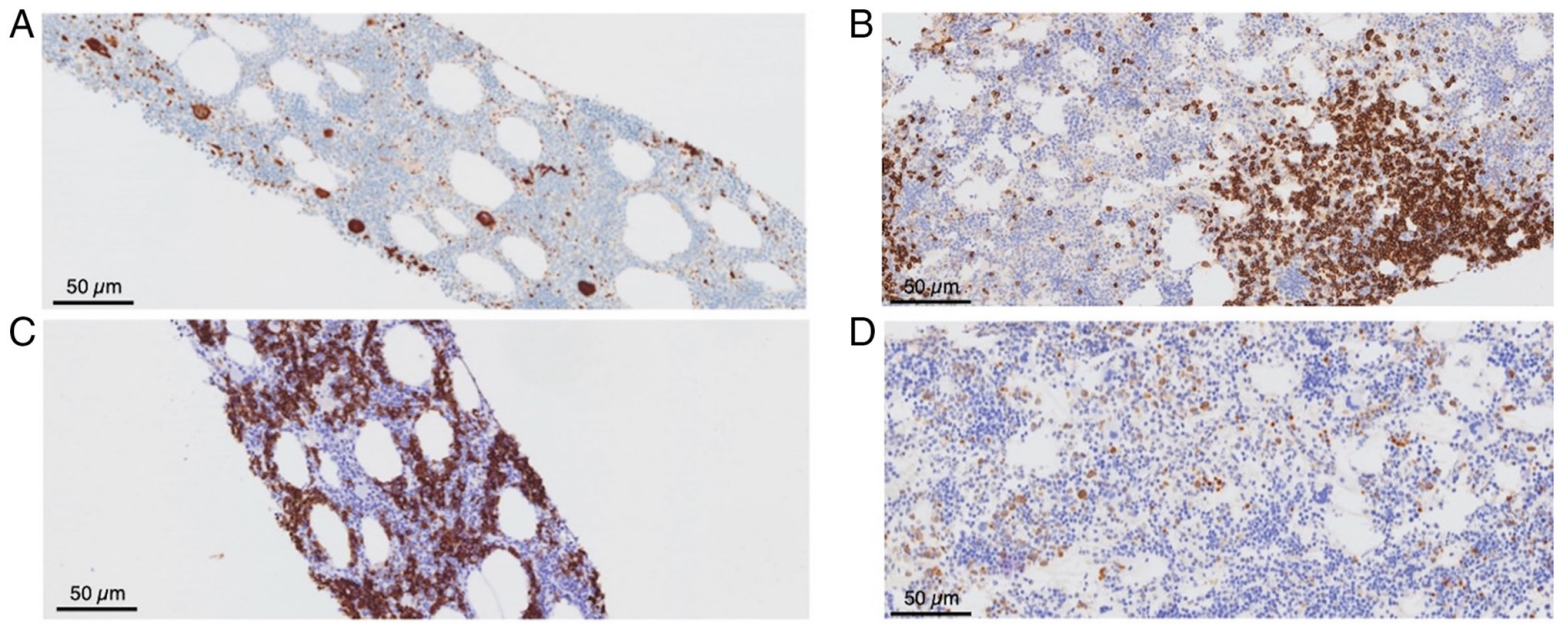
Immunohistochemical staining demonstrating trilineage hematopoiesis within the retroperitoneal mass. (A) CD61 highlights clusters of megakaryocytes. (B) CD20 illustrates lymphoid aggregates composed of polyclonal B-lymphocytes. (C) CD71 highlights erythroid precursors. (D) MPO illustrates staining of a subset of myeloid cells. Magnification, x200 for all images.

Due to critical comorbidities, the patient was deemed a poor surgical candidate for mass resection. Interventional radiologists performed a core needle biopsy. A histopathological examination was performed using 3.5-µm-thick sections obtained from formalin-fixed, paraffin-embedded tissue. The specimens were fixed in 10% neutral-buffered formalin at 18-25˚C for 2-4 h. Hematoxylin and eosin staining (Leica Biosystems) was performed at ambient temperature for 45 min. The stained sections were examined using a light microscope (Olympus BX43; Olympus Corporation). A histopathological analysis revealed adipose tissue infiltrated by trilineage hematopoietic elements, without evidence of dysplasia, lipoblasts, or spindle cell morphology (Fig. 2). In addition, immunohistochemical analysis was performed on 3.5-µm-thick formalin-fixed, paraffin-embedded tissue sections using an automated Leica BOND platform (Leica Biosystems) with a 3,3'-diaminobenzidine (DAB) chromogenic detection system. Antigen retrieval was performed on the Leica BOND platform under manufacturer-recommended conditions: epitope retrieval using ER2 buffer (alkaline, Leica Biosystems) for CD20, and ER1 buffer (citrate-based, Leica Biosystems) for MPO. For intracellular antigens, permeabilization was achieved using the automated Leica BOND protocol with manufacturer-provided proprietary reagents. Blocking was performed using Leica BOND system blocking reagents according to the manufacturer's instructions. Primary antibodies against CD20 (clone L26, cat. no. PA0200, Leica Biosystems, ready-to-use) and MPO (clone 59A5, cat. no. PA0491, Leica Biosystems, ready-to-use) were incubated on the Leica BOND platform at ambient temperature for 20 and 5 min, respectively. CD71 and CD61 immunostains were performed at an external reference laboratory (Quest Diagnostics) using their validated protocols. A Leica BOND polymer-based detection system (HRP-conjugated) was used for visualization with DAB chromogen. Sections were counterstained with hematoxylin (Leica Biosystems) at ambient temperature for 10 min. Immunostained sections were examined using a light microscope (Olympus BX43; Olympus Corporation). Immunohistochemical staining was positive for CD45, MPO, CD71 and CD61, and negative for atypical markers (Fig. 3). Flow cytometry analysis did not demonstrate evidence of a hematologic malignancy; however, the corresponding raw plot data were not available for inclusion, as only the finalized clinical report was accessible at the time of review (data not shown). These findings were consistent with EAML, a rare, benign lesion typically composed of mature fat and hematopoietic tissue. Serum protein electrophoresis and globulin fractions were also reviewed and no notable findings were obtained; there was no evidence of a monoclonal process.

The patient remained clinically stable during hospitalization and was discharged on the 8th hospital day with outpatient follow-up planned with hematology/oncology. Gastroenterology was consulted with plans for upper and lower endoscopy as an outpatient to further evaluate his weight loss and gastrointestinal symptoms. Given his notable comorbidities, an echocardiogram was also recommended for outpatient follow-up to assess cardiac function. Unfortunately, the patient succumbed approximately 2 weeks following discharge of causes not related to his medical condition.

## Discussion

EAMLs are rare, benign tumors composed of mature adipose tissue and trilineage hematopoietic elements (3). Of note <100 cases have been reported in the literature, with the majority occurring in the retroperitoneum or presacral space (3). Other documented extra-adrenal sites include the mediastinum, liver, spleen, lungs, kidneys and paravertebral regions (3). The mean age at onset is 61 years and these lesions affect females more than males (6). Lesion size varies, although most range from 4 to 10 cm (7). The mass in the patient described herein measured ~9 cm, placing it within the upper size range and contributing to radiological concern for malignancy. Although numerous patients are asymptomatic, larger lesions may cause symptoms related to mass effect, including abdominal pain, early satiety or urinary obstruction (8).

Unlike adrenal myelolipomas, which are more frequently encountered, EAMLs are far less common and are often discovered incidentally during imaging for unrelated concerns (8). They are often identified on CT scans or MRI as heterogeneous, fat-containing masses (8). However, their radiological appearance can closely mimic malignancies, such as liposarcoma. The differential diagnosis for fat containing retroperitoneal masses is broad and carries critical management implications. Well differentiated liposarcoma is the most important entity to exclude, as it is the most common primary retroperitoneal fat containing malignancy and can appear radiographically indistinguishable from EAML on a CT scan or MRI. Histologically, liposarcomas are characterized by the presence of lipoblasts and zones of cellular atypia, which were absent in the patient in the present study. Other fat-containing lesions in the differential include angiomyolipoma, retroperitoneal teratoma, retroperitoneal lipoma and extramedullary hematopoiesis (EMH)-related masses (9). EMH and EAML may share both imaging and histopathological features; however, EMH typically presents as a multifocal, poorly circumscribed lesion without macroscopic fat, whereas EAML is usually well encapsulated with prominent adipose tissue. Angiomyolipoma, while more commonly renal in origin, can occasionally arise in extra-renal retroperitoneal locations and should also be considered. Technetium-99m sulfur colloid scintigraphy has been described in prior case reports as an adjunctive imaging tool that can help differentiate EAML from liposarcoma, as myelolipomatous tissue takes up the tracer, while liposarcoma does not (6). Nevertheless, tissue diagnosis via core needle biopsy remains the definitive standard, as was performed in the case presented herein.

The pathogenesis of EAMLs is not yet fully understood. Proposed mechanisms include the metaplasia of mesenchymal or reticuloendothelial cells, or development from ectopic adrenal tissue (5). Although in the patient described herein Cushing's disease was treated a number of years prior, some literature suggests that chronic ACTH stimulation, such as that observed in untreated or prolonged Cushing's disease, may play a role in myelolipoma formation via mesenchymal metaplasia (5,10). Although in the patient in the present study Cushing's disease was surgically treated and biochemically controlled for decades, prior prolonged exposure to elevated ACTH levels before definitive treatment may have contributed to tumor development. Reports of EAMLs in patients with remote endocrine disorders are uncommon, rendering this case noteworthy.

In the patient in the present study, pathological analysis revealed mature adipose tissue with trilineage hematopoiesis, consistent with EMH, and no signs of atypia, malignant transformation or necrosis, as previously described (8). Grossly, EAMLs are well-circumscribed, with a lobulated cut surface composed of yellow fat and red-brown marrow-like areas (8). Flow cytometry did not reveal any clonal populations (data not shown). Although EAMLs are considered benign and nonfunctional, the presence of EMH raises a critical consideration of the possibility of an underlying marrow disorder. EMH is typically associated with conditions such as myelofibrosis, chronic hemolysis, or thalassemia, but can also be observed in rare benign tumors such as this one (1,11).

In the patient described herein, there were no laboratory or clinical signs of a hematological disorder. Still, hematology/oncology follow-up was appropriate to ensure that no occult pathology was missed and to monitor for any evolving signs of marrow dysfunction. Several prior case reports have described retroperitoneal EAML initially misdiagnosed as liposarcoma, highlighting the importance of histopathological confirmation before committing to a treatment strategy, as the two entities carry vastly different prognoses and operative implications (3,4,6). Once the diagnosis of retroperitoneal EAML is confirmed, management is guided by lesion size, symptom burden and the presence of complications. Conservative management with surveillance imaging is appropriate for asymptomatic lesions. Surgical resection is generally indicated for masses >6 cm in size, symptomatic lesions, those causing mass effect on adjacent structures, such as ureteral compression, or cases with persistent diagnostic uncertainty (8). In the patient in the present study, the mass measured 6.3x7.0x8.8 cm with apparent ureteral encasement features that would conventionally favor surgical resection. However, given his critical comorbidities, operative intervention was deemed prohibitively high-risk, and a tissue-first approach via CT-guided biopsy was appropriately pursued. The present case report illustrates that in patients who are poor surgical candidates, percutaneous core needle biopsy can establish a definitive diagnosis and guide conservative management without the risks of major surgery. Management is generally conservative once the diagnosis is confirmed and the patient remains asymptomatic (8). Surgery is reserved for symptomatic lesions, diagnostic uncertainty, or rapid growth (8). Prognosis is excellent, with no malignant potential described (8).

The present case report highlights how EAML can mimic malignancy and reinforces the importance of integrating imaging, pathology, and clinical context when evaluating fat-containing retroperitoneal masses. It also reflects how EMH, while often benign, can sometimes serve as a clue to underlying hematologic disease and should prompt further evaluation when identified in unexpected locations. Additionally, it underscores the potential role of prior endocrine disorders, such as Cushing's disease, as a contributing factor in the pathogenesis of EAML, even when the primary condition has been long controlled.

In the patient described herein, the experienced weight loss was felt to be multifactorial. Contributing factors included a decreased appetite from the mass effect of the retroperitoneal lesion and age-related deconditioning. Thyroid function was well controlled. It is worth noting that while Cushing's disease is classically associated with weight gain, the disease of the patient had been treated decades prior, and his endocrine profile reflecting adrenal insufficiency rather than active hypercortisolism, rendering it an unlikely contributor. Cardiac evaluation included an EKG stable to prior, with no clinical symptoms of cardiac disease; echocardiography was recommended as an outpatient study to exclude cardiomyopathy. Endoscopy was similarly deferred to the outpatient setting to rule out an occult gastrointestinal source contributing to weight loss. These represent limitations of this case, as a complete outpatient workup could not be fulfilled given the unexpected passing of the patient.

In conclusion, the present case report illustrates the diagnostic challenge of distinguishing extra-adrenal myelolipoma from malignancy based on imaging alone. EAML should be considered in the differential for fat-containing retroperitoneal lesions, particularly in patients with a history of chronic endocrine disorders such as Cushing's disease, which may play a role in pathogenesis. Histopathology remains essential for definitive diagnosis, and management is typically conservative unless the lesion is symptomatic or diagnostic uncertainty persists.

## Data Availability

The data generated in the present study may be requested from the corresponding author.
